# Global end-diastolic volume is an important contributor to increased extravascular lung water in patients with acute lung injury and acuterespiratory distress syndrome: a multicenter observational study

**DOI:** 10.1186/2052-0492-2-25

**Published:** 2014-04-01

**Authors:** Tadashi Kaneko, Yoshikatsu Kawamura, Tsuyoshi Maekawa, Takashi Tagami, Toshiaki Nakamura, Nobuyuki Saito, Yasuhide Kitazawa, Hiroyasu Ishikura, Manabu Sugita, Kazuo Okuchi, Hiroshi Rinka, Akihiro Watanabe, Yoichi Kase, Shigeki Kushimoto, Hiroo Izumino, Takashi Kanemura, Kazuhide Yoshikawa, Hiroyuki Takahashi, Takayuki Irahara, Teruo Sakamoto, Yuichi Kuroki, Yasuhiko Taira, Ryutarou Seo, Junko Yamaguchi, Makoto Takatori, on behalf of the PiCCO Pulmonary Edema Study Group

**Affiliations:** Advanced Medical Emergency and Critical Care Center (AMEC3), Yamaguchi University Hospital, 1-1-1 Minamikogushi, Ube, Yamaguchi, 755-8505 Japan; Department of Emergency and Critical Care Medicine, Aidu Chuo Hospital, 1-1 Tsuruga, Aiduwakamatsu, Fukushima, 965-8611 Japan; Department of Emergency and Critical Care Medicine, Nippon Medical School Hospital, 1-1-5 Sendagi, Bunkyo-ku, Tokyo, 113-8603 Japan; Intensive Care Unit, Nagasaki University Hospital, 1-7-1 Sakamoto, Nagasaki, 852-8501 Japan; Department of Emergency and Critical Care Medicine, Nippon Medical School Chiba Hokusoh Hospital, 1715 Kamagari, Inzai-shi, Chiba, 270-1694 Japan; Department of Emergency and Critical Care Medicine, Kansai Medical University, 10-15 Fumizono-cho, Moriguchi City, Osaka, 570-8506 Japan; Department of Emergency and Critical Care Medicine, Faculty of Medicine, Fukuoka University, 7-45-1 Nanakuma, Jonan-ku, Fukuoka City, Fukuoka, 814-0180 Japan; Department of Emergency and Critical Care Medicine, Juntendo University Nerima Hospital, 3-1-10 Takanodai, Nerima-ku, Tokyo, 177-8521 Japan; Department of Emergency and Critical Care Medicine, Nara Medical University, 840 Shinjo-cho, Kashihara, Nara, 634-8521 Japan; Emergency and Critical Care Medical Center, Osaka City General Hospital, 2-13-22 Miyakojima Hondori, Miyakojima, Osaka, 534-0021 Japan; Department of Critical Care Medicine, Jikei University School of Medicine, 3-19-18 Nishi-shinbashi, Minato-ku, Tokyo, 105-8471 Japan; Division of Emergency Medicine, Tohoku University Graduate School of Medicine, 1-1 Seiryo-machi, Aiba-ku, Sendai, 980-8574 Japan; Advanced Emergency and Critical Care Center, Kansai Medical University Takii Hospital, 10-15 Fumizono-machi, Moriguchi City, Osaka, 570-8507 Japan; Emergency and Critical Care Medicine, National Hospital Organization Disaster Medical Center, 3256 Midori-cho, Tachikawa-shi, Tokyo, 190-0014 Japan; Shock Trauma and Emergency Medical Center, Tokyo Medical and Dental University Hospital, 1-5-45 Yushima, Bunkyo-ku, Tokyo, 113-8519 Japan; Department of Intensive Care Medicine, Saiseikai Yokohamashi Tobu Hospital, 3-6-1 Shimosumiyosi, Tsurumi-ku, Yokohama City, Kanagawa, 230-8765 Japan; Department of Emergency and Critical Care Medicine, Nippon Medical School Tama Nagayama Hospital, 1-7-1 Nagayama, Tama-shi, Tokyo, 206-8512 Japan; Department of Emergency and Critical Care Medicine, Kurume University School of Medicine, 67 Asahi-machi, Kurume-shi, Fukuoka, 830-0011 Japan; Department of Emergency and Critical Care Medicine, Social Insurance Chukyo Hospital, 1-1-10 Sanjo, Mimami-ku, Nagoya City, Aichi, 457-8510 Japan; Department of Emergency and Critical Care Medicine, St. Marianna University School of Medicine, 2-16-1 Sugao, Miyamae, Kawasaki, Kanagawa, 216-8511 Japan; Department of Anesthesia, Kobe City Medical Center General Hospital, 2-2-1 Minatojimaminamimachi, Chuo-ku, Kobe City, Hyogo, 650-0046 Japan; Division of Emergency and Critical Care Medicine, Department of Acute Medicine, Nihon University School of Medicine, 30-1 Oyaguchi-Kamimachi, Itabashi-ku, Tokyo, 173-8610 Japan; Department of Anesthesia and Intensive Care, Hiroshima City Hospital, 7-33 Motomachi, Naka-ku, Hiroshima-shi, Hiroshima, 730-8518 Japan

**Keywords:** Pulmonary edema, Extravascular lung water, Multivariate regression analysis, Global end-diastolic volume, Acute lung injury, Acute respiratory distress syndrome

## Abstract

**Background:**

Extravascular lung water (EVLW), as measured by the thermodilution method, reflects the extent of pulmonary edema. Currently, there are no clinically effective treatments for preventing increases in pulmonary vascular permeability, a hallmark of lung pathophysiology, in patients with acute lung injury/acute respiratory distress syndrome (ALI/ARDS). In this study, we examined the contributions of hemodynamic and osmolarity factors, for which appropriate interventions are expected in critical care, to EVLW in patients with ALI/ARDS.

**Methods:**

We performed a subgroup analysis of a multicenter observational study of patients with acute pulmonary edema. Overall, 207 patients with ALI/ARDS were enrolled in the study. Multivariate regression analysis was used to evaluate the associations of hemodynamic and serum osmolarity parameters with the EVLW index (EVLWI; calculated as EVLW/Ideal body weight). We analyzed factors measured on the day of enrollment (day 0), and on days 1 and 2 after enrollment.

**Results:**

Multivariate regression analysis showed that global end-diastolic volume index (GEDVI) was significantly associated with EVLWI measured on days 0, 1, and 2 (*P* = 0.002, *P* < 0.001, and *P* = 0.003, respectively), whereas other factors were not significantly associated with EVLWI measured on all 3 days.

**Conclusions:**

Among several hemodynamic and serum osmolarity factors that could be targets for appropriate intervention, GEDVI appears to be a key contributor to EVLWI in patients with ALI/ARDS.

**Trial registration:**

University Hospital Medical Information Network (UMIN) Clinical Trials Registry UMIN000003627.

## Background

Pulmonary edema is classified into cardiogenic and non-cardiogenic types. Cardiogenic pulmonary edema is caused by hydrostatic factors (e.g., volume overload and/or pulmonary hypertension caused by inadequate cardiac function), whereas non-cardiogenic pulmonary edema is caused by vascular permeability, which is also known as permeability pulmonary edema [1]. Both forms of pulmonary edema are associated with increased extravascular lung water. Extravascular lung water (EVLW) can be measured by the transpulmonary thermodilution technique and is strongly correlated with actual lung weight at autopsy and at donor surgery, which reflects pulmonary edema in humans [2–4].

Acute lung injury/acute respiratory distress syndrome (ALI/ARDS) is one of the most common types of permeability pulmonary edema. Among patients with ALI/ARDS, an increase in EVLW is associated with increased risk of mortality [5]. In most types of cardiogenic pulmonary edema, increases in EVLW are caused by volume overload by fluid infusion in the presence of inadequate cardiac function. By comparison, the risk factors for permeability pulmonary edema in ALI/ARDS, for which appropriate interventions are expected in critical care, are still unknown. In terms of the pathophysiology of ALI/ARDS, the most important treatment target is to inhibit the increase in pulmonary permeability; currently, however, there are no effective agents and/or ventilator strategies for use in clinical settings. Our preliminary study showed that hemodynamic and osmolarity parameters were risk factors for hydrostatic and permeability pulmonary edema [6]. This is important because hemodynamic and osmolarity factors could be controlled by the physician. In this context, the aim of this study was to identify hemodynamic and/or osmolarity risk factors for an increase of extravascular lung water index (EVLWI) that could be controlled in clinical settings as possible treatment targets for ALI/ARDS.

## Methods

### Study design

Between March 2009 and August 2011, we conducted a multicenter observational study to assess the clinical diagnosis of cardiogenic and non-cardiogenic pulmonary edema, which were assessed using the transpulmonary thermodilution technique [7]. The protocol of this subgroup analysis was approved by the institutional review boards of all participating institutions, and written informed consent was provided by each patient’s next of kin. The investigation was registered with the University Hospital Medical Information Network (UMIN) Clinical Trials Registry (UMIN-CTR ID: UMIN000003627).

### Patients

The inclusion and exclusion criteria were the same as those in our previous study [7]. Patients aged >15 years old were eligible if they required mechanical ventilation, had a PaO_2_/FiO_2_ ratio of ≤300 mmHg, had bilateral infiltration on chest X-ray images, and were monitored by transpulmonary thermodilution. Exclusion criteria included the following: >5 days since the onset of respiratory failure, chronic lung disease, history of a pulmonary operation, pulmonary embolism, severe peripheral artery disease, cardiac index (CI) of <1.5 L/min · m^2^ with cardiogenic shock, lung contusion or burns, or the attending physician considered the patient as inappropriate for this study. Data from 207 patients with ALI/ARDS that was diagnosed by three or more experts in intensive care, respiratory, and cardiology, with appropriate clinical findings (e.g., medical history, physiological findings, radiological data, laboratory data, and echocardiography), were analyzed in this study.

### Measurements

Transpulmonary thermodilution was performed using the PiCCO® monitor (Pulsion Medical Systems SE, Munich, Germany) in all institutions. A 4- or 5-Fr femoral arterial thermistor-tipped catheter (PC2014L16 or PV2015L20; Pulsion Medical Systems SE) was inserted into the patient and was connected to the PiCCO® plus or PiCCO® 2 monitor. The PiCCO® monitor uses the single-thermal indicator technique to calculate volumetric parameters. A bolus of 15 mL of cold saline is injected through a central venous catheter, and thermal variation is detected by the thermistor at the tip of the arterial catheter. Cardiac output is calculated using the Stewart-Hamilton method. The mean transit time and the exponential downslope time of the transpulmonary thermodilution curve are also calculated by this system. Each value provided by the PiCCO® monitor was calculated as the mean of three bolus injections of normal saline [8]. Each value was indexed according to the patient’s height and ideal body weight using the following formula: Body weight (kg) = 50 + 0.91 (Height (cm) – 152.4) for males and body weight = 45.5 + 0.91 (Height (cm) – 152.5) for females, as previously reported [9].

The transpulmonary thermodilution technique calculates the CI, global end-diastolic volume index (GEDVI) (corresponding to global diastolic cardiac volume in relation to preload), and EVLWI (a quantitative assessment of pulmonary edema [2–4] calculated as Extravascular lung water/Ideal body weight). Analysis of the arterial pulse contour by the PiCCO® monitor also provides other parameters, including continuous cardiac output and the stroke volume variation (SVV), which is the percentage in respiratory-induced stroke volume variation in relation to preload.

We conducted multivariate linear regression analyses to identify which of the following hemodynamic and osmolarity parameters were associated with EVLWI: GEDVI, SVV, and central venous pressure (CVP) as preload parameters, systemic vascular resistance index (SVRI) as afterload parameter, CI as hemodynamic parameter, and albumin (ALB) and serum osmolarity (OSM) as serum osmolarity parameters. OSM was calculated using the formula: OSM = 2(Na + K) + BS/18 + BUN/2.8, where Na = sodium concentration (mmol/L), K = potassium concentration (mmol/L), BS = blood sugar concentration (mg/dL), and BUN = blood urea nitrogen concentration (mg/dL). These data were obtained on the day of enrollment (day 0) and on days 1 and 2 after enrollment. OSM was only determined on day 0.

### Statistical analysis

Statistical analyses were performed using SPSS software version 16.0 (IBM, Armonk, NY, USA). Univariate analyses were performed using Student’s *t* test. Multivariate regression analyses were performed with a stepwise procedure to identify the statistically significant factors associated with the dependent variable, EVLWI. The independent variables were age, sex (‘male’ was treated as a variable), GEDVI, SVV, CVP, SVRI, CI, ALB, and OSM. Correlation analysis was performed using Pearson’s correlation test. The analyses were performed for each day of measurement. All statistical analyses were considered significant at *P* < 0.05.

## Results

The characteristics and parameters measured in the 207 patients are summarized in Table 1. The mean age was 66.7 years old. There were 134 males and 73 females, and 61.8% had sepsis. The mean APACHE II score, SOFA score, PaO_2_/FiO_2_ ratio, and lung injury score [10] were 23.4, 10.7, 150.5 mmHg, and 2.3, respectively. The 28-day mortality rate was 40.6%. The comparison between survivor and non-survivor, age, APACHE II score, SOFA score, CVP on day 1, SVV on day 2, and CVP on day 2 were significantly different.

**Table 1:** **Patient characteristics**

Variable	All cases	Survivor	Non-survivor	***P*** value
*n*	207	123	84	
Age (years)	66.7 ± 16.8	63.9 ± 17.6	70.7 ± 14.9	*0.004*
Male	134 (64.7%)	77 (62.6%)	57 (67.9%)	0.437
Sepsis, yes	128 (61.8%)	70 (56.9%)	58 (69.0%)	0.078
APACHE II score	23.4 ± 8.1	21.9 ± 7.8	25.6 ± 8.1	*0.001*
SOFA score	10.7 ± 3.6	10.0 ± 3.2	11.8 ± 3.7	*<0.001*
PaO_2_/FiO_2_ ratio (mmHg)	150.5 ± 70.9	155.8 ± 70.2	142.8 ± 71.6	0.195
Lung injury score	2.3 ± 0.6	2.3 ± 0.6	2.3 ± 0.6	0.702
28-day mortality rate	84 (40.6%)	0 (0%)	84 (100%)	-
Day 0				
EVLWI (mL/kg)	18.5 ± 6.8	18.5 ± 6.9	18.5 ± 6.7	0.981
GEDVI (mL/m^2^)	816.8 ± 205.7	823.0 ± 211.8	807.5 ± 197.2	0.595
SVV (%)	15.7 ± 6.9	15.4 ± 7.3	16.1 ± 6.4	0.472
CVP (mmHg)	10.2 ± 5.3	9.8 ± 4.9	10.7 ± 5.8	0.221
CI (L/min · m^2^)	3.5 ± 1.3	3.6 ± 1.2	3.3 ± 1.3	0.171
SVRI (dyn · s · cm^−5^ · m^2^)	1,805 ± 866	1,716 ± 707	1,932 ± 1,043	0.083
ALB (g/dL)	2.6 ± 0.7	2.6 ± 0.7	2.6 ± 0.7	0.509
OSM (mOsm/L)	304.7 ± 16.7	303.5 ± 15.6	306.4 ± 18.1	0.227
PEEP (cmH_2_O)	8.7 ± 4.7	8.8 ± 4.7	8.5 ± 4.8	0.720
Day 1				
EVLWI (mL/kg)	17.7 ± 7.4	17.3 ± 7.2	18.4 ± 7.8	0.280
GEDVI (mL/m^2^)	821.4 ± 222.5	828.8 ± 222.3	810.1 ± 223.7	0.561
SVV (%)	14.2 ± 7.6	14.0 ± 8.7	14.6 ± 5.7	0.581
CVP (mmHg)	10.6 ± 4.8	9.9 ± 4.4	11.7 ± 5.2	*0.007*
CI (L/min · m^2^)	3.5 ± 1.2	3.6 ± 1.1	3.3 ± 1.3	0.119
SVRI (dyn · s · cm^−5^ · m^2^)	1,880 ± 852	1,865 ± 873	1,902 ± 824	0.767
ALB (g/dL)	2.5 ± 0.6	2.5 ± 0.6	2.4 ± 0.6	0.304
PEEP (cmH_2_O)	8.9 ± 5.2	9.0 ± 5.1	8.9 ± 5.5	0.934
Day 2				
EVLWI (mL/kg)	16.4 ± 7.4	15.7 ± 6.4	17.7 ± 8.8	0.077
GEDVI (mL/m^2^)	858.3 ± 238.2	844.7 ± 237.2	880.2 ± 239.7	0.315
SVV (%)	12.4 ± 5.9	10.9 ± 5.5	14.7 ± 5.8	*<0.001*
CVP (mmHg)	10.4 ± 5.0	9.6 ± 4.3	11.7 ± 5.7	*0.004*
CI (L/min · m^2^)	3.7 ± 1.3	3.7 ± 1.3	3.7 ± 1.3	0.920
SVRI (dyn · s · cm^−5^ · m^2^)	1,791 ± 733	1,791 ± 733	1,791 ± 733	0.977
ALB (g/dL)	2.5 ± 0.6	2.5 ± 0.6	2.5 ± 0.6	0.880
PEEP (cmH_2_O)	8.5 ± 5.0	8.5 ± 4.9	8.5 ± 5.3	0.947

Values are means ± standard deviation or *n* (%). *P* value: survivor vs. non-survivor. The italicized values are statistically significant. *APACHE* acute physiology and chronic health evaluation, *SOFA* sequential organ failure assessment, *EVLWI* extravascular lung water index, *GEDVI* global end-diastolic volume index, *SVV* stroke volume variation, *CVP* central venous pressure, *CI* cardiac index, *SVRI* systemic vascular resistance index, *ALB* serum albumin, *OSM* calculated serum osmotic pressure (OSM = 2(Na + K) + BS / 18 + BUN / 2.8, where Na = sodium concentration (mmol/L), K = potassium concentration (mmol/L), BS = blood sugar concentration (mg/dL), and BUN = blood urea nitrogen concentration (mg/dL)), *PEEP* positive end-expiratory pressure.

Table 2 shows the results of the multivariate regression analysis performed using the stepwise procedure. GEDVI and CI were significantly associated with EVLWI on day 0 (*P* = 0.002 and *P* = 0.023, respectively). GEDVI and sex were significantly associated with EVLWI on day 1 (*P* < 0.001 and *P* = 0.015, respectively). Sex, GEDVI, and SVRI were significantly associated with EVLWI on day 2 (*P* = 0.002, *P* = 0.003, and *P* = 0.049, respectively).

**Table 2:** **Results of multivariate regression analysis with EVLWI as the dependent variable**

Variable	***P*** value	Regression coefficient (95% confidence interval)	Standard regression coefficient
Day 0			
GEDVI	0.002	0.009 (0.004, 0.015)	0.272
CI	0.023	−1.045 (−1.941, −0.148)	−0.195
*R* ^2^ = 0.076	0.003		
Day 1			
GEDVI	<0.001	0.011 (0.006, 0.017)	0.330
Sex (male)	0.015	−3.045 (−5.500, −0.590)	−0.188
*R* ^2^ = 0.135	<0.001		
Day 2			
Sex (male)	0.002	−4.161 (−6.764, −1.557)	−0.256
GEDVI	0.003	0.008 (0.003, 0.014)	0.251
SVRI	0.049	0.002 (0.000, 0.003)	0.158
Age	0.051	0.074 (0.000, 0.148)	0.162
*R* ^2^ = 0.181	<0.001		

Regression analyses were performed using the stepwise procedure. *EVLWI* extravascular lung water index, *GEDVI* global end-diastolic volume index, *CI* cardiac index, *SVRI* systemic vascular resistance index.

**Figure 1:**
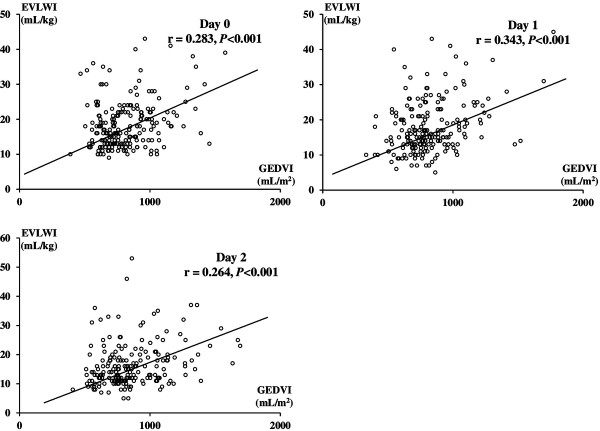
**Correlation between extravascular lung water index (EVLWI) and global end-diastolic volume index (GEDVI) in each day.** Pearson’s correlation coefficients in days 0, 1, and 2 were 0.283, 0.343, and 0.264, respectively (*P* < 0.001 in each day).

Figure 1 shows the results of Pearson’s correlation test between GEDVI and EVLWI. Pearson’s correlation coefficients in day 0, 1, and 2 were 0.283, 0.343, and 0.264, respectively (*P* < 0.001 in each day).

## Discussion

The results of multivariate regression analysis suggested that in patients with ALI/ARDS, EVLWI was strongly associated with GEDVI measured for 3 days after enrollment in this study. The management of hemodynamic status by diuresis and limited fluid infusion in patients with cardiogenic pulmonary edema is already well established [11]. By contrast, the management of permeability pulmonary edema in ALI/ARDS is still not well defined. Because the severity of cardiogenic pulmonary edema is related to the patient’s hemodynamic status, then logically, the hemodynamic monitoring technique should follow this pathophysiology. However, in patients with ALI/ARDS, permeability pulmonary edema is not directly proportional to hemodynamic parameters [7]. In the present study, EVLWI was used to quantify pulmonary edema and was strongly associated with GEDVI measured on all 3 days, suggesting that appropriate control of the circulating blood volume is an important factor in the management of not only cardiogenic edema but also permeability pulmonary edema. Consequently, GEDVI could also be a target for the management of ALI/ARDS.

Options for fluid management in patients with ALI/ARDS have already been assessed in a randomized controlled trial, where a conservative fluid strategy to control blood volume, by avoiding excessive water balance, improved the oxygenation index, lung injury score, and ventilator-free days but did not affect mortality [12]. Another study showed that mortality was increased in patients who did not receive adequate fluid management (an initial fluid bolus and avoidance of a positive water balance for 1 week) [13]. Although EVLWI and GEDVI were not evaluated in those studies, adequate blood volume management may improve lung function. GEDVI is an index of preload regulation. Although the management of blood volume improves lung function [12, 13], target GEDVI levels have not been defined. In fact, the target GEDVI should be conservative, with limited fluid infusion, to avoid worsening of EVLWI. However, if fluid management is too conservative, it may result in inadequate blood volume that can cause organ ischemia. Therefore, an appropriate target of GEDVI should be established [14]. Moreover, recent studies have shown that pulmonary permeability can be monitored and quantified using the transpulmonary thermodilution technique [7, 15–17]. The target GEDVI level may need to be adjusted according to the severity of pulmonary permeability by evaluating the cutoff value for increases in EVLWI, although this needs to be investigated in future studies. However, in tradition, other preload parameters (CVP, SVV, etc.) did not show the target value; therefore, it is unclear whether the target GEDVI could be shown or not.

In a preliminary study, serum osmolarity factors, such as decreased ALB and increased OSM, were positively associated with EVLWI [6], which was not confirmed in this study. In patients with ALI/ARDS, it was reported that albumin infusion and diuresis did not improve mortality, but did improve oxygenation [18, 19]. These preliminary results suggest that serum osmolarity factors may decrease EVLWI, although this was not statistically significant in our study. The type of fluid being used to control blood volume may be an important factor in patients with severely increased pulmonary permeability. Current data suggest that fluid resuscitation of albumin for severe sepsis and septic shock is recommended over hydroxyethyl starch [20], which can be used in the early phase of ALI/ARDS resuscitation without worsening EVLWI [21]. Although ALB and OSM were not significantly associated with EVLWI in the present study, the type of fluid used for resuscitation should be assessed in future studies.

In the present study, most of the significant parameters were from hemodynamics (GEDVI, CI, and SVRI). However, sex was other significant parameter on days 2 and 3. These results showed that females had advantage for EVLWI increase. Previous study showed that gender affected the normal range of GEDVI [22]; therefore, there is a possibility that gender could affect EVLWI, because of strong relationship between EVLWI and GEDVI in our data.

Our data showed a 40.6% 28-day mortality rate, and the average APACHE II score was 23.4. The average age of the non-survivor cases was significantly older (survivor vs. non-survivor 63.9 vs. 70.7, *P* = 0.004), and the non-survivor cases had tendency of high GEDVI and EVLWI on later days. It might be a result of fluid management protocol, which was not defined in this study. These results also indicated that there might be better fluid management.

Our study has several limitations. First, it was conducted as a secondary subgroup study, which means that the results should be confirmed in a prospective study. Second, the severity of pulmonary permeability was not considered, and the classification of pulmonary permeability may alter the results.

Multivariate analysis showed that GEDVI was the most predictor for an increase in EVLWI. These results are consistent with those of earlier fluid management studies in patients with ALI/ARDS [12, 13]. To aid the clinical management of patients with ALI/ARDS, we suggest that EVLWI and GEDVI should be measured to examine their clinical relevance as potential therapeutic targets. This possibility should be confirmed in a future study.

## Conclusions

EVLWI as a quantitative marker of pulmonary edema is strongly associated with GEDVI in patients with ALI/ARDS. Therefore, GEDVI may be a therapeutic target to help control extravascular lung water. Prospective studies are needed to examine the effects of targeting GEDVI for improving EVLWI and clinical outcomes in patients with ALI/ARDS.

## Abbreviations

ALB: Serum albumin
ALI: Acute lung injury
ARDS: Acute respiratory distress syndrome
APACHE: Acute physiology and chronic health evaluation
CI: Cardiac index
CVP: Central venous pressure
EVLWI: Extravascular lung water index
GEDVI: Global end-diastolic volume index
OSM: Calculated serum osmotic pressure
PEEP: Positive end-expiratory pressure
SOFA: Sequential organ failure assessment
SVV: Stroke volume variation.
